# Simulating Properties of In Vitro Epithelial Cell Morphogenesis 

**DOI:** 10.1371/journal.pcbi.0020129

**Published:** 2006-10-06

**Authors:** Mark R Grant, Keith E Mostov, Thea D Tlsty, C. Anthony Hunt

**Affiliations:** 1 University of California San Francisco/University of California Berkeley Joint Graduate Group in Bioengineering, University of California Berkeley, Berkeley, California, United States of America; 2 Department of Anatomy, University of California San Francisco, San Francisco, California, United States of America; 3 Department of Pathology, University of California San Francisco, San Francisco, California, United States of America; 4 Department of Biopharmaceutical Sciences, Biosystems Group, University of California San Francisco, San Francisco, California, United States of America; University of Virginia, United States of America

## Abstract

How do individual epithelial cells (ECs) organize into multicellular structures? ECs are studied in vitro to help answer that question. Characteristic growth features include stable cyst formation in embedded culture, inverted cyst formation in suspension culture, and lumen formation in overlay culture. Formation of these characteristic structures is believed to be a consequence of an intrinsic program of differentiation and de-differentiation. To help discover how such a program may function, we developed an in silico analogue in which space, events, and time are discretized. Software agents and objects represent cells and components of the environment. “Cells” act independently. The “program” governing their behavior is embedded within each in the form of axioms and an inflexible decisional process. Relationships between the axioms and recognized cell functions are specified. Interactions between “cells” and environment components during simulation give rise to a complex in silico phenotype characterized by context-dependent structures that mimic counterparts observed in four different in vitro culture conditions: a targeted set of in vitro phenotypic attributes was matched by in silico attributes. However, for a particular growth condition, the analogue failed to exhibit behaviors characteristic of functionally polarized ECs. We solved this problem by following an iterative refinement method that improved the first analogue and led to a second: it exhibited characteristic differentiation and growth properties in all simulated growth conditions. It is the first model to simultaneously provide a representation of nonpolarized and structurally polarized cell types, and a mechanism for their interconversion. The second analogue also uses an inflexible axiomatic program. When specific axioms are relaxed, growths strikingly characteristic of cancerous and precancerous lesions are observed. In one case, the simulated cause is aberrant matrix production. Analogue design facilitates gaining deeper insight into such phenomena by making it easy to replace low-resolution components with increasingly detailed and realistic components.

## Synopsis

To gain new insights into how normal and abnormal epithelial cell (EC) morphogenesis occurs, Grant and colleagues designed, built, and studied a series of discrete event analogues capable of mimicking epithelial growth characteristics in four different culture conditions. The analogues use independent software agents and objects to represent cells and the two environment components. “Cells” interact with local components using an axiomatic decisional process deduced from experimental in vitro observations. During simulations, “cells” form stable structures that mimic counterparts in cell cultures: a set of targeted in vitro phenotypic attributes is matched by the analogue's phenotype. However, the foundational analogue failed to exhibit a behavior characteristic of functionally polarized ECs in stable structures. Iterative refinement solved the problem: the improved analogue is the first model to simultaneously provide a representation of nonpolarized and structurally polarized cell types, and a mechanism for their interconversion. Inflexible axiom application is essential to simulate normal attributes. Selectively changing an axiom or relaxing its application caused growths strikingly characteristic of cancerous and precancerous lesions. Gaining deeper insight into such phenomena can be achieved by replacing low-resolution components with increasingly detailed and realistic components.

## Introduction

How do individual cells organize into multicellular tissues? O'Brien et al. [1] have convincingly argued that the morphogenetic behavior of epithelial cells (ECs) is guided by two distinct elements: an intrinsic differentiation program that drives formation of lumen-enclosing monolayers, and transient de-differentiation that allows the monolayer to be remodeled. The intrinsic differentiation program is argued to be a consequence of an innate drive for each cell to achieve three surface types: free (contact with a luminal space), lateral (contact with another cell), and basal (contact with and attachment to matrix). Traditionally, such broad-ranging conceptual models have been validated by the absence of contradiction within the accumulated experimental wet-lab evidence. Experimentation in silico on synthetic simulation models of the type described herein may be an effective new alternative.

Assume that an intrinsic differentiation program exists. What biological principles are represented? How are those principles connected simultaneously to molecular level events and systemic, phenotypic attributes? A proven way to understand phenomena and to challenge conceptual models such as that of O'Brien et al. is to build a device—a synthetic analogue—that exhibits those phenomena. We report here on doing just that. We have built, studied, and improved in silico devices that instantiate the above conceptual model. They exhibit several of the key behaviors described by O'Brien et al. Understanding the operating principles and mechanisms responsible for normal EC growth and morphogenesis, along with the conditions that can lead to abnormalities, is expected to accelerate the development of treatments for diseases such as autosomal dominant polycystic kidney disease and carcinomas, and enable improved designs of artificial devices that utilize ECs.

Several mathematical models of EC growth are capable of matching observations taken from surface culture, the most common of the various culture conditions. One of the more mature of these models describes cells in terms of elastic spheres, with differential binding affinity to cells and matrix, and force-driven cell division [2]. Another predicts EC repair behavior in response to varying levels of calcium [3]. Both models incorporate matrix-dependent cell survival; they have demonstrated their usefulness in predicting EC growth properties under particular surface culture conditions. However, they were not intended to represent other fundamental behaviors such as the de novo production of extracellular matrix. These models were not designed to represent EC behavior in conditions other than surface culture, in part because they rely on assumptions that are incompatible with observations of growth in different culture conditions. Without considerable reengineering, they cannot be used to mimic EC attributes in more complex culture conditions. We need models of EC growth and morphogenesis that are capable of mimicking phenotypic attributes of ECs grown under a variety of culture conditions. These models are expected to be more useful in predicting outcomes in the diverse conditions that can occur in vivo and in vitro. Studying the behaviors of several related models under a range of conditions is needed in order to identify and eliminate those that exhibit an acceptable behavior, such as a growth attribute in one condition, but not in others.

The complexity of the processes involved in forming a structure as simple as the cysts formed in vitro pose multiple challenges to the modeler. Foundational models are needed, from which more detailed descendents can easily be created and extended, coupled with a rigorous approach for adding the required detail. A middle-out modeling strategy, first suggested by Brenner [4] and later detailed by Noble [5], has been proposed for building models of complex biological systems. The approach specifies beginning at the level for which sufficient biological data exists to support model construction. We have adapted the approach to achieve the above-stated objectives.

**Figure 1:**
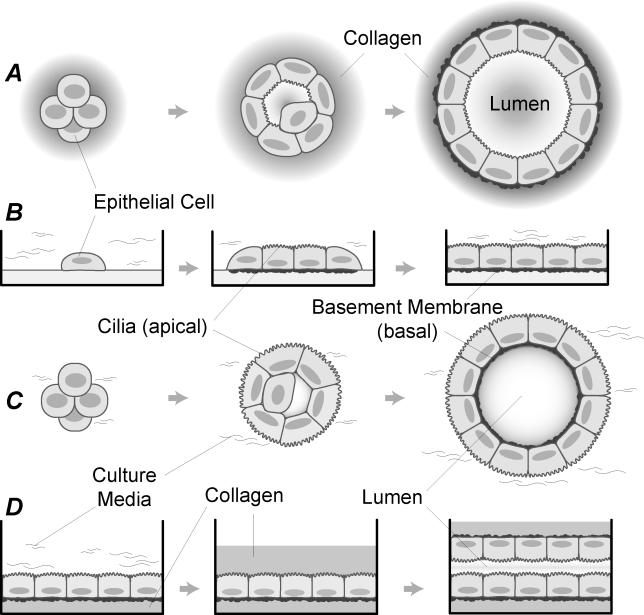
EC Growth Characteristics in Four Different In Vitro Culture Conditions (Cross-Section) For each culture condition, development typically starts with one or several ECs. (A) Cell division, apoptosis, and shape change over a period of several days in embedded culture often leads to a stable, lumen-containing cyst formed by a single layer of ECs. (B) ECs plated on a layer of collagen (surface culture) typically generate a stable, uniform monolayer. (C) Inverted cysts (cell polarity relative to that in (A) is inverted) form in suspension culture, with matrix deposited on the inside of the cyst. (D) Lumens are frequently formed in collagen overlay experiments.

Observations of EC growth and morphogenesis in different environments (Figure 1) document that morphology in vitro depends on both the structure and composition of the environment external to the cells. In 3-D embedded cultures, formation of stable, self-enclosed monolayers (cysts) is the dominant morphological characteristic (Figure 1A). In suspension cultures, ECs also form cysts, but the ECs adopt an inverted polarity, depositing basement membrane on the inside of the cyst (Figure 1C). In surface culture, ECs typically form a coherent monolayer covering the entire matrix surface (Figure 1B). Overlay of a monolayer of ECs with Collagen I, however, induces a morphological response, in which ECs form stable cyst-like structures with a central lumen (Figure 1D). Each is a characteristic outcome of the growth of a number of different EC types in vitro, suggesting a shared morphological program [6–8]. Together, these characteristics form our initial set of targeted attributes. They implicate the presence and relative locations of cells, matrix, and matrix-free (and cell-free) space (hereafter, simply free space) as being powerful determinants of EC behavior. We therefore speculated that mechanistic principles that simply distinguish between these components would be the appropriate level for a foundational model, and that is what we describe in this report.

**Figure 2:**
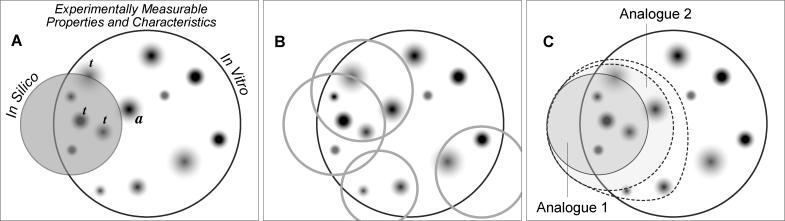
A Process for Iterative Refinement of Biological Models An abstract Venn-like diagram illustrates the behavior similarities (depicted as overlapping sets of features) of in silico and in vitro models. (A) The shaded circle contains the set of observable, measurable attributes (qualities, properties, etc.) of an in silico model such as Analogue 1 or 2. The larger circle is the infinite set of possible observable, measurable attributes of an in vitro model that is being viewed or studied from a particular perspective, with attention focused on selected aspects of the system. ***a***: Each small shaded domain represents the results of wet-lab experiments intended to measure a specific in vitro property or characteristic. The degree of shading illustrates different levels of experimental and measurement uncertainty. ***t***: a member of the set of *targeted* in vitro attributes that the in silico model is expected to mimic. (B) This sketch illustrates that it may take many different in silico models, possibly drawn from different classes of models, to obtain even partial behavior coverage of the in vitro system. (C) Illustrated is the systematic, sequential extension of the in silico analogue's measurable attributes to improve coverage. The original shaded set can illustrate Analogue 1. The first dashed circle illustrates expanding the original set of targeted attributes by including one or more additional properties. The established analogue (Analogue 1) fails to generate the required outcomes, resulting in its invalidation. A copy of the established analogue can be iteratively refined (without losing or breaking the original behaviors) by adding new details as needed. The goal is to have all of the expanded set of targeted attributes (original plus expanded coverage) adequately represented by a new analogue, such as Analogue 2, represented by the first dashed circle. The process can be repeated indefinitely by adding attributes to the target set and then adding new capabilities to Analogue 2 as before. With each extension, the goal is to have the expanded set of targeted attributes all adequately represented. If the goal cannot be achieved without “breaking” the original model, then either a new model or multiple separate models, as in (B), may be required.

Models of EC morphogenesis constructed using currently available experimental information will be incomplete. To continue to be useful, these models will need to be changed and adapted as new experimental information becomes available. Strategies for refining biological system models have been proposed. For example, Palsson [9] proposes an iterative model-building process that relies upon specification of constraints on biological activities and the identification of models that can satisfy those constraints. However, the method focuses on the molecular details of specific cellular processes, and many such processes are known to contribute to morphogenic behaviors such as those targeted above. Nevertheless, we have taken the concepts and extended them to models capable of exhibiting multiple systemic phenotypic attributes. We present and use that strategy for the iterative refinement of models with complex in silico phenotypes. Figure 2 illustrates the envisioned relationship between a model's phenotype and that of the in vitro biological referent. Pictured are two overlapping (but not intersecting) sets. Each set contains results of experiments that focused on specific, measurable attributes. When the two sets of measures are similar, such as in silico and in vitro formation of cysts and other structures in particular environments, then there may also be useful similarities in their generative mechanisms, and these can be explored by iterative testing (in silico experimentation) and model refinement.

Coordination of in vitro and in vivo experiments is a proven means of advancing knowledge of the organism of interest. Similarly, coordination of in silico and in vitro experimentation may prove to be a powerful means of more efficiently generating useful new knowledge about in vitro systems and, ultimately, their in vivo and patient referents. Realization of this idea requires that simulation models be constructed in such a way as to be readily modifiable to represent additional biological detail, as required. In order to mimic growth characteristics in new experimental observations, the in silico cell components will need to be flexible and adaptable.

In this report, we describe simulation models of cell growth and morphogenesis that can mimic (validate against) targeted phenotypic attributes of ECs grown under four different culture conditions. They are constructed using an object-oriented, discrete event, discrete environment approach in which cell behavior is governed by a set of axioms. The foundational model—Analogue 1—is designed to have nine in silico capabilities that are needed to enable easy, iterative refinement and extension. After validation of Analogue 1 against the initial set of targeted attributes, we identified a behavior that did not correspond to known in vitro observations. Exhibiting the proper behavior was added to the set of targeted attributes. We then developed a revised model—Analogue 2—to have a more acceptable behavior and retain the validated behaviors exhibited by Analogue 1. The result is a model of EC morphogenesis that functions under a wide range of simulation conditions, providing descriptions of simulated cell behavior in those conditions that may have in vitro counterparts. The model is distinct from previous models of epithelial morphogenesis because it simultaneously provides a representation of nonpolarized and structurally polarized cell types, and a mechanism for their interconversion. We also describe the consequences of selectively disrupting Analogue 2 mechanisms, and identify additional phenotypic attributes of the type that will need to be targeted by future analogues, descendents of Analogue 2.

## Results

To avoid confusion and clearly distinguish in vitro components from corresponding simulation components, such as “cells,” “matrix,” “cyst,” and “lumen,” we use small capital letters when referring to simulation components and phenotypic attributes.

### Simulating EC Behavior

To keep the foundational model as simple as possible yet capable of simulating growth in a variety of environments, we placed the initial representation of mechanism close to the simulation of the systemic phenomena of interest, the initial set of targeted attributes. We did this by devising a set of simple axioms that could be used by independent cells to mimic EC behavior in a range of simulated environments. *Axiom* emphasizes that computer programs are mathematical, formal systems, and the initial mechanistic premises in our simulations are analogous to axioms in formal systems. For our analogues, an axiom is an assumption about what conclusion can be drawn from what precondition (assumed true based on the preponderance evidence) for the purposes of further analysis or deduction, and for developing the analogue system: during simulation, if a specific precondition is met, then a specific action will follow.

In a particular in vitro growth situation, many cellular processes work together in ways that give rise to an effective mandate that all normal ECs appear to follow in that situation. Each mandate is assumed to be a consequence of the interoperation of genetics, development, and environmental factors. For the foundational analogue, it was our goal to represent the essence of these apparent mandates using simple axioms. They are placeholders for more detailed representations that can be developed later. We refer to such analogues as axiomatic models (AMs). Because specific processes contribute differently to the apparent mandate that emerges, we assumed that most axioms would reflect involvement of two or more of the known capabilities and functions of ECs grown in vitro. For example, structure stabilization in polarizing environments and the fact that ECs adhere to one another during growth contributed to the development of several axioms, but not in equivalent ways. A more detailed analogue that separately represents such functions can be called a functionality-based model (FBM). The importance of the relationships between the two model types and between axioms and functions is presented in the Discussion section.

We needed the above set of axioms to provide informed predictions of EC behavior in a diverse range of simulated conditions. In vitro observations are most often made using surface cultures, but useful information can be derived about EC behavior in other environments such as when they are grown within, rather than on top of, media containing matrix. Less commonly used in vitro culture systems such as suspension, embedded, and overlay cultures provided most of the information used for axiom construction. For example, the composition of the environment directly adjacent to a cell in a growing cyst in embedded culture conditions varies considerably from one location to another. Also, cells inside a growing cyst typically undergo apoptosis, whereas those found on the outside do not.

**Figure 3:**
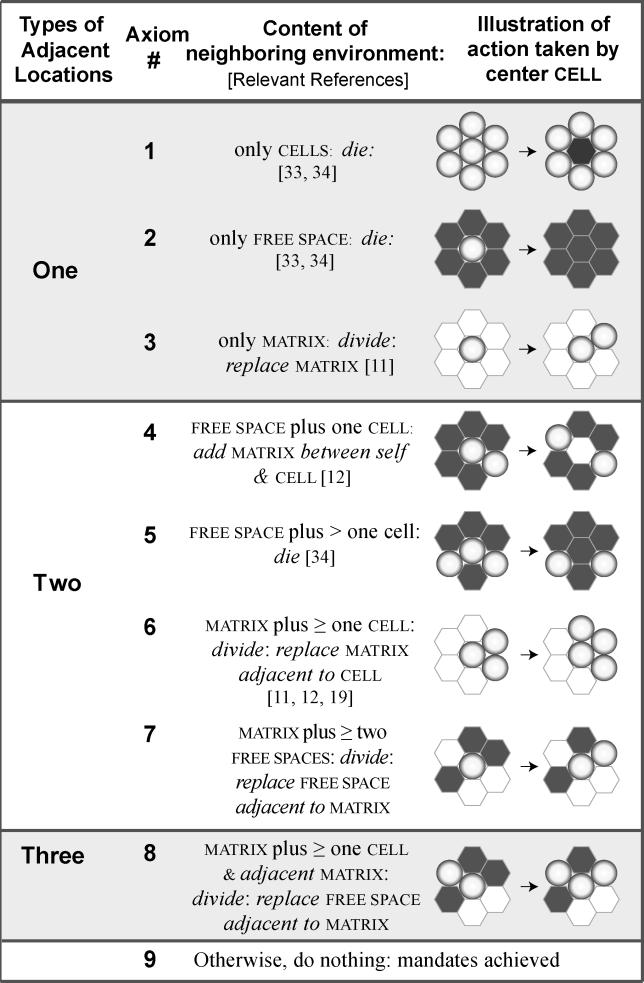
Axioms Developed from In Vitro Observations of EC Behavior in Different Culture Conditions They specify the action a cell will take given a precondition: the composition and relative arrangement of components in its local neighborhood, directly adjacent to the cell. The neighborhood can contain one, two, or three types of object (cell, matrix, and free space). Black grid spaces represent free space; circular, shaded spheres represent cells; and white grid spaces represent matrix. When multiple neighborhood arrangements meet the requirements for placement of a daughter cell, one is selected at random. If a particular environment configuration does not meet one of the listed conditions for division or death, the cell does nothing.

**Figure 4:**
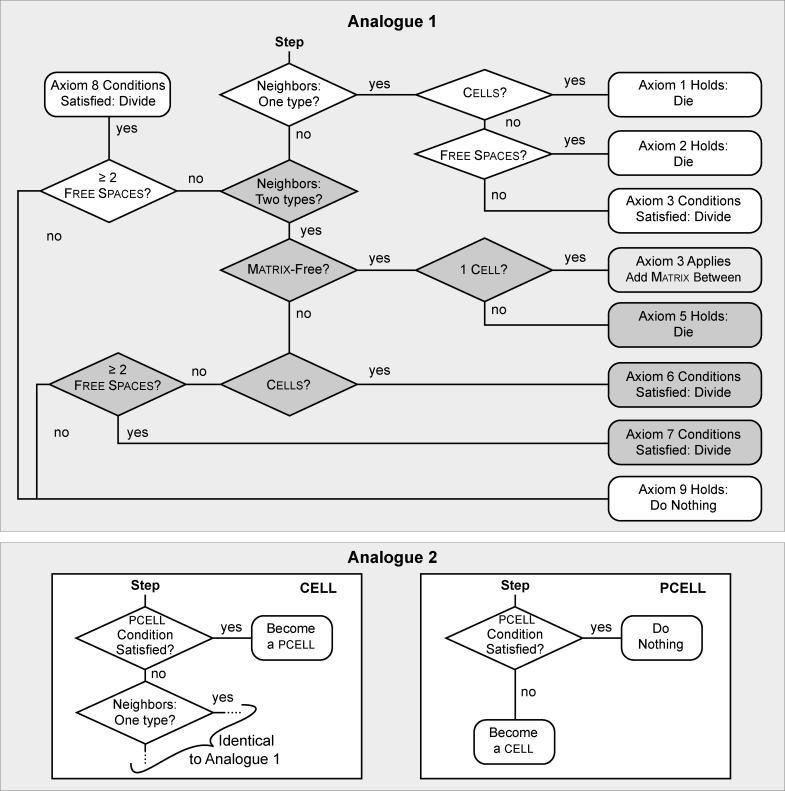
The Flow of Decisionmaking—Axiom Application—by Each Cell During Simulation During each simulation cycle, each cell has an option to act (the order of selection is randomized each cycle). For Analogue 1 the basic options are die, divide, move, and add matrix, or do nothing. Analogue 2 (lower boxes) provides three additional options: transition to pcell, remain a pcell, or transition to cell status. When its turn arrives, the cell assesses the status of its local neighborhood (types of components and their relative locations). That information establishes the precondition for axiom application. The diagram abstracts up from many different particular simulation runs of the analogue and represents possible chains of inference that any one cell may follow in any given simulation cycle. For each precondition, one axiom applies and one action results. During the next simulation cycle, the process repeats, independent of what occurred in any prior cycle.

The set of axioms derived based on observations of cell behavior in these diverse culture systems is summarized in Figure 3. Some details on the creation of these axioms are provided in Figures S1 and S2. Figure 4 is a decisional flow diagram showing how the axioms are used during simulation. During each simulation cycle, a cell assesses its current condition—the adjacent components and their arrangement in the local neighborhood. It then determines which axiom condition is satisfied and takes the mandated action. Each cell has only one action option.

### Phenotypic Attributes of Analogue 1

**Figure 5:**
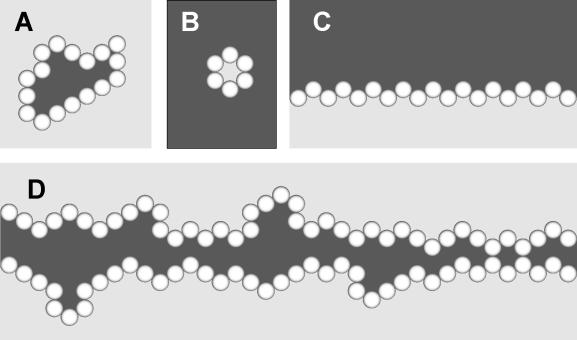
Examples of Analogue 1 Outcomes in Four Simulated Environments Spheres represent cells; black space represents free space or a simulated lumen space; a light shaded space represents matrix. (A) Stable cysts formed in simulated embedded culture. (B) A stable, inverted cyst formed in simulated suspension culture. (C) A stable monolayer formed in simulated surface culture. (D) A stable lumen formed in simulated overlay culture.

The axioms provided a foundation for our foundational model of epithelial morphogenesis, hereafter referred to as Analogue 1. We tested the model by comparing simulated outcomes with observations of EC behavior in four standard in vitro environments. We observed 1) uniform, stable monolayers in simulated surface culture, 2) stable cyst-like structures (cysts) with an internal lumen-like region (lumen) in simulated embedded culture, 3) cysts with an internal matrix region in simulated suspension culture, and 4) lumens lined by cells in simulated overlay culture (Figure 5). Additional details are provided in Figure S3, but they are not essential to the explanation of experimental results described in subsequent sections.

We explored the behavior of Analogue 1 in 2-D simulated embedded culture. The EC line MDCK (Madin-Darby canine kidney) II embedded in type I collagen exhibits clonal growth in two stages to form stable cysts [7,10]. The first stage involves repeated rounds of cell division, occurring during the first two days of culture. Second is the formation of a central lumen after three to four rounds of cell division. Lumen formation is accompanied by an increase in cell number and cyst diameter between day two and day seven. Thereafter, cyst size plateaus and the cysts survive in culture with little apparent change for several weeks. Simulations of Analogue 1 exhibited five properties and characteristics (PCs) that by observation were strikingly similar to those observed for MDCK II in embedded culture. 1) After 2–2.5 simulated days of growth, free space appears; thereafter it accumulates within the expanding cluster of cells. This is the beginning of a lumen-like central region for what becomes a cyst. Lumen formation is observed to occur in vitro by as early as day 2 [7]. 2) Cyst expansion always arrested and thereafter no changes occurred (Figures S4 and S5) [7]. 3) Stable cysts contain a lumen lined by a single layer of cells ( Figure 5 A). We observed this condition in all simulations. 4) Cell division and cell death continue after the initiation of lumen formation (Figure S5), a characteristic observed during growth of MCF10A cells [11], another ductal EC line, as well as during growth of MDCK cells [12]. 5) The variation in cell number per mature cyst is similar to in vitro observations (Figure S5).

Cystogenesis during in vitro suspension culture begins with the formation of aggregates of two to ten cells. Thereafter, over approximately ten days, an “inverted” cyst (Figure 1C) forms and expands. During the process, basement membrane components, including Collagen IV and Laminin I, accumulate in the lumen and line the inner surface of the cyst [7]. Some matrix is apparently produced de novo, and has been observed to occur between two adhering ECs in suspension culture [13]. This secreted matrix likely influences cell survival and may induce neighboring cells to polarize in a common direction. We limited matrix production to one situation: when a cell has only one other cell neighbor (Axiom 4). This abstract behavioral specification is sufficient to enable formation of small, stable cysts in simulated suspension culture (Figure 5B).

Analogue 1 formed coherent monolayers in simulated surface culture. ECs grown on Collagen I typically generate a simple monolayer that covers the entire surface [14]. Most cell division takes place on the outer edge of an expanding colony [15]. As one would expect from the axioms, monolayer formation always occurred on flat matrix surfaces in 2-D simulation (Figure 5C).

The final, targeted attribute was lumen formation in simulated overlay culture. Overlay of a MDCK cell monolayer with Collagen I induces a morphological response mediated by integrins [16]. The binding of integrins on the apical surface to Collagen I sets off a cascade of events that results in the generation of lumen spaces completely surrounded by cells [6]. These events include migration, division, apoptosis, and shape changing. After stabilization, most, if not all cells, have cell–cell and cell–matrix attachments and border common luminal spaces. Simulation of overlay culture with Analogue 1 resulted in the formation of structures similar to those just described (Figure 5D): regions of free space are formed surrounded by single layers of cells, each bordering matrix. These results demonstrate the first five of the capabilities described under Materials and Methods.

### Analogue Invalidation

Having demonstrated that Analogue 1 exhibits the targeted PCs, we were poised to move forward, using the approach in Figure 2 to create an improved model. The next step was to select the additional attribute to add to the set of targeted in vitro attributes. We could select one for which there was no Analogue 1 counterpart, or one for which the Analogue 1 attribute appeared inconsistent with in vitro observations. The following examples are of four of the attributes that we considered.

Example 1) In embedded culture in vitro there is occasional expansion of cysts even after a distinct monolayer surrounding a central lumen has formed [7]. That expansion may be a consequence of cell division. Analogue 1 exhibits no corresponding behavior because division is not an option for cells that have already satisfied the axioms in Figure 3. Example 2) Some stable cysts are quite aspherical, more wrinkled and puckered than the one in Figure 5A (for examples, see Figure S4). Such shapes are rarely observed in vitro. Example 3) Not all cells placed in embedded culture in vitro form lumen-filled cysts. A few stable cell clusters apparently have no lumens at all; the reason is not known. Example 4) Cells differentiate during in vitro growth. The last stage in this process is thought to be adoption of apical–basolateral polarity. This change occurs after several days in culture, as ECs reorganize their internal structures in response to change in the adjacent environment. Once polarity is established, they have the ability to directionally transport molecules between apical and basolateral surfaces, and those surfaces become histologically distinct. These polarized cells and their undifferentiated predecessors are distinct from each other [1] and respond to local interventions quite differentially. With respect to the Example 4), Analogue 1′s axioms and logic are designed to allow for differentiation, but it occurs below the level of resolution of a cell. Consequently, the cells comprising a stable cyst will not respond differently to a change in the adjacent environment than will a cell at the beginning or in the early stages of in silico growth: they use the same axioms and follow the same logic. Behaviors expected of cells having adopted apical–basolateral polarity will not be observed.

**Figure 6:**
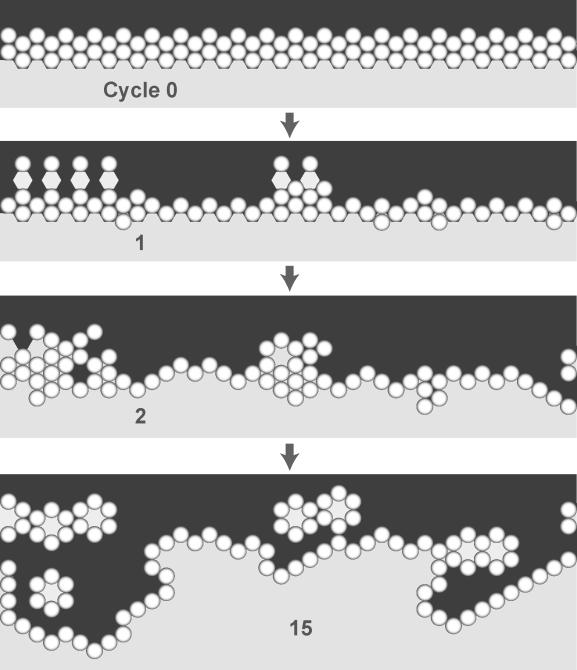
An Invalid Response from Analogue 1 The sketched screenshots show the induced growth response of a monolayer of cells in simulated surface culture following placement of a second layer of cells above the first. A black space represents free space or a simulated lumen space; a light shaded space represents matrix. All the cells in a stable monolayer in vitro are polarized and so are expected to be relatively unresponsive to the newly added cells. First, a stable monolayer of cells was formed. The simulation was stopped. A layer of cells was placed on top of the monolayer (top: cycle 0). The simulation was restarted. End of cycle 1: many of the added cells have “died;” growth and development of cells in the original monolayer have been stimulated; encroachment of cells into the matrix below has started. Cycle 2: remodeling is under way. Cycle 15: after considerable remodeling, a stable structure along with several inverted cysts has formed.

We elected to use Example 4) as a basis for moving toward an improved analogue. We added a new attribute to the set of targeted attributes: a cell that is a member of a stable structure must respond differently to some changes in its adjacent neighborhood, relative to cells at an earlier growth stage. The former must be more resistant to taking an action (one, such as division, which would be associated with a less differentiated state in the referent). Simulation experiments were designed to document the behavior of Analogue 1 under conditions where apical–basolateral polarization is expected to influence cell behavior. A stable monolayer was constructed upon which another monolayer was placed (Figure 6). Simulation resulted in proliferation in the underlying monolayer and remodeling of the matrix. No similar in vitro observations have been reported, nor would they be expected. We also observed the formation of matrix-filled cysts above the remodeled surface (Figure 6, Step 15). One of the results of apical–basolateral polarization is that the cells become insensitive to the presence of other cells at their apical surface. Faced with the revised set of targeted attributes, the proliferation behavior in Figure 6 invalidates Analogue 1. One strategy to avoid such behavior is to refine the model such that some aspect of apical–basolateral polarity is represented in the improved analogue so that its behaviors are more consistent with apical–basolateral polarity.

### Analogue 2

**Figure 7:**
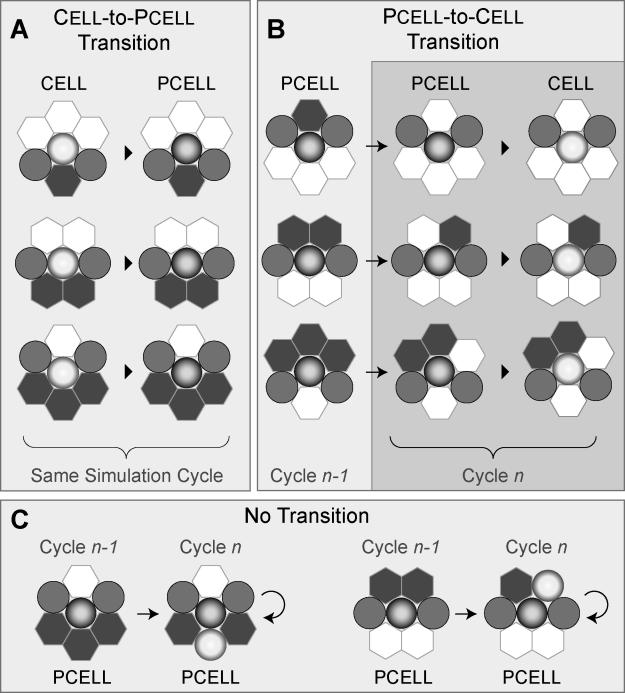
Analogue 2: Preconditions Determining Transitions between Cell and Pcell, or Lack Thereof The decisional sequences followed during simulation are illustrated in Figures 4 and 8. (A) During a simulation cycle each cell assesses its neighborhood and consults the axioms to determine if it qualifies to become a pcell (Figure 4, lower left). Any one of the three arrangements shown qualifies. If qualified, the transition occurs immediately and it enters the next simulation cycle as a pcell. (B) During a simulation cycle each pcell (that was a pcell in the preceding simulation cycle) assesses its neighborhood and consults the axioms to determine if it qualifies to stay a pcell (Figure 4, lower right). The three arrangements shown (middle column) do not qualify. If not qualified (to remain a pcell), the transition to cell occurs immediately. (C) Illustrated are changes to a pcell's local neighborhood that do not meet any of the preconditions for a pcell-to-cell transition; it remains a pcell.

**Figure 8:**
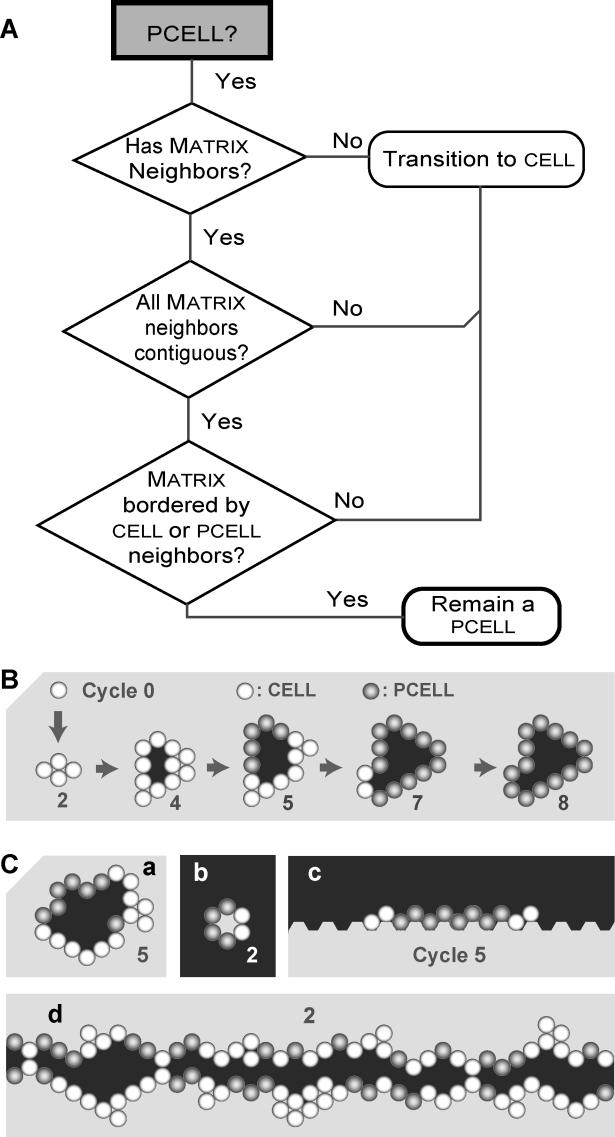
Retention of Pcell Status (A) Given a pcell precondition, if three conditions in sequence are also met, as illustrated by the decisional flow diagram, then pcell status is retained. Otherwise, the pcell transitions to cell. (B) Sketched are sequential screen shots taken during a typical Analogue 2 simulation for simulated embedded culture. Spheres represent cells and pcells; pcells are shaded darker. A black space represents free space or a simulated lumen space; a light shaded space represents matrix. The first evidence of pcells is at the end of simulation cycle 5. Cycle 8: a stable structure has formed; only pcells are present. (C) An example of an Analogue 2 outcome from each of the four simulated environments, comparable to the Analogue 1 outcome in Figure 5. The status of the developing structures in the four simulated environments at simulation cycle 2 or 5 is shown. Each contains a mixture of cells and pcells: **a**: a developing cyst in simulated embedded culture; **b**: a cyst in simulated suspension culture; **c**: monolayer formation in simulated surface culture; and **d**: lumen formation in simulated overlay culture.

To achieve the desired new analogue, several options were available. One was to abandon Analogue 1 and start over. The preferred option (that strives for capabilities 6–8 in Materials and Methods) was to modify Analogue 1 so that it could exhibit the new attribute. To demonstrate the viability of iterative model refinement along with the flexibility and reusability capabilities (see Materials and Methods), we followed the latter approach. The simplest strategy was to maintain the existing resolution and to introduce a new component, a pcell, to represent an EC exhibiting apical–basolateral polarity. Having four components rather than three required specifying the preconditions under which a cell-to-pcell transition would occur, how such a transition would take place in silico, and the conditions for reversion from pcell to cell. Examples of conditions under which these transitions would occur are summarized in Figure 7. In the case of MDCK cells, apical–basolateral polarity is associated with the presence of neighboring ECs separating a well-defined matrix surface from an adjacent region free of matrix or cells. We assumed that such a configuration is necessary and sufficient for polarization, and identified local neighborhood arrangements that would lead to cell-to-pcell transition (Figure 7A), as described under Materials and Methods. These arrangements were selected with the expectation that in mandating the pcell transition they would significantly inhibit the invalidating behavior in Figure 6 by maintaining a pcell state when neighboring free space is replaced by cells or other pcells (Figure 7C). The preconditions for a return to a cell state are illustrated in Figure 7B (for additional detail, see Figure S6), and reflect observations that matrix exerts a depolarizing response when present on both sides of a polarized monolayer (as in overlay culture), and that ECs, even if polarized, will migrate into gaps in matrix. These observations were used to construct a decisionmaking process to govern pcell behavior (Figure 8A).

Any change to a simulation model, such as those just described, may compromise the original model's ability to generate a previously satisfactory outcome. Avoidance of such problems was a motivation for specification of the reusability capability described under Materials and Methods. Our first task was thus to confirm that the revisions did not significantly compromise Analogue 2′s ability to satisfy the original set of four targeted attributes. Figure 8B shows an example of growth in simulated embedded conditions, demonstrating that stable cyst formation still occurs with Analogue 2 and demonstrating transition of cells to pcells. The outcomes from Analogue 2 are similar to those produced by Analogue 1 for the four targeted culture conditions (Figure 8C). For the simulated surface and suspension conditions, the results were identical. The results for the two analogues in simulated, embedded, and overlay culture conditions were not identical. However, they were as similar as one would expect from two independent repetitions of the same in vitro experiment conducted in the same laboratory: they were experimentally indistinguishable (Figure S5). Taken together, these results demonstrate the value of designing analogues to exhibit the flexibility and reusability capabilities.

**Figure 9:**
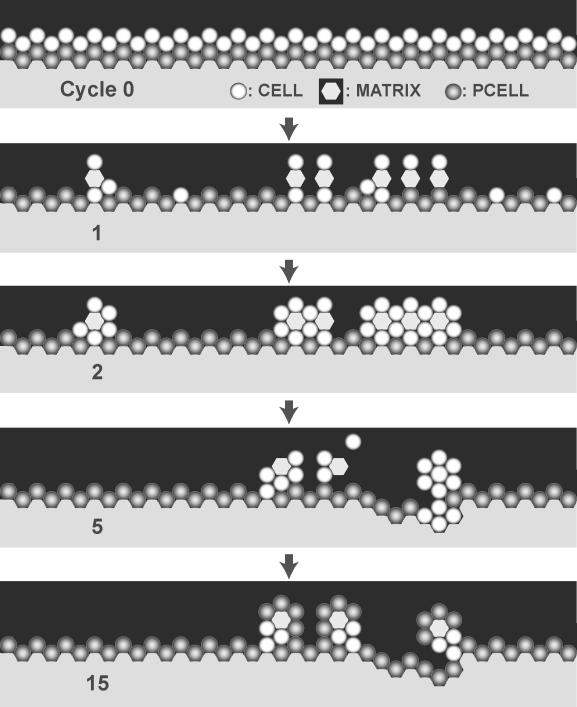
An Appropriate Response by Analogue 2 An appropriate response is demonstrated by Analogue 2 in the previously invalidating experiment described in Figure 6. This experiment determined that inclusion of pcells in Analogue 2 provides some resistance to the unacceptable, dramatic behavior of Analogue 1. Sketched are screen shots taken during a typical simulation. Pcells are shaded darker than cells. Spaces are shaded as defined in Figure 8. First, a stable monolayer of pcells was formed. The simulation was stopped. A layer of cells was placed on top of the pcell monolayer (top: cycle 0). The simulation was restarted. End of cycle 1: many of the added cells have “died,” and a few of the original pcells have transitioned to cell status. Cycle 5: some minor remodeling of the original layer of matrix has occurred; compare with cycle 2 in Figure 6. Cycle 15: a stable structure has formed dominated by pcells; some inverted cysts remain above the pcell monolayer.

**Figure 10:**
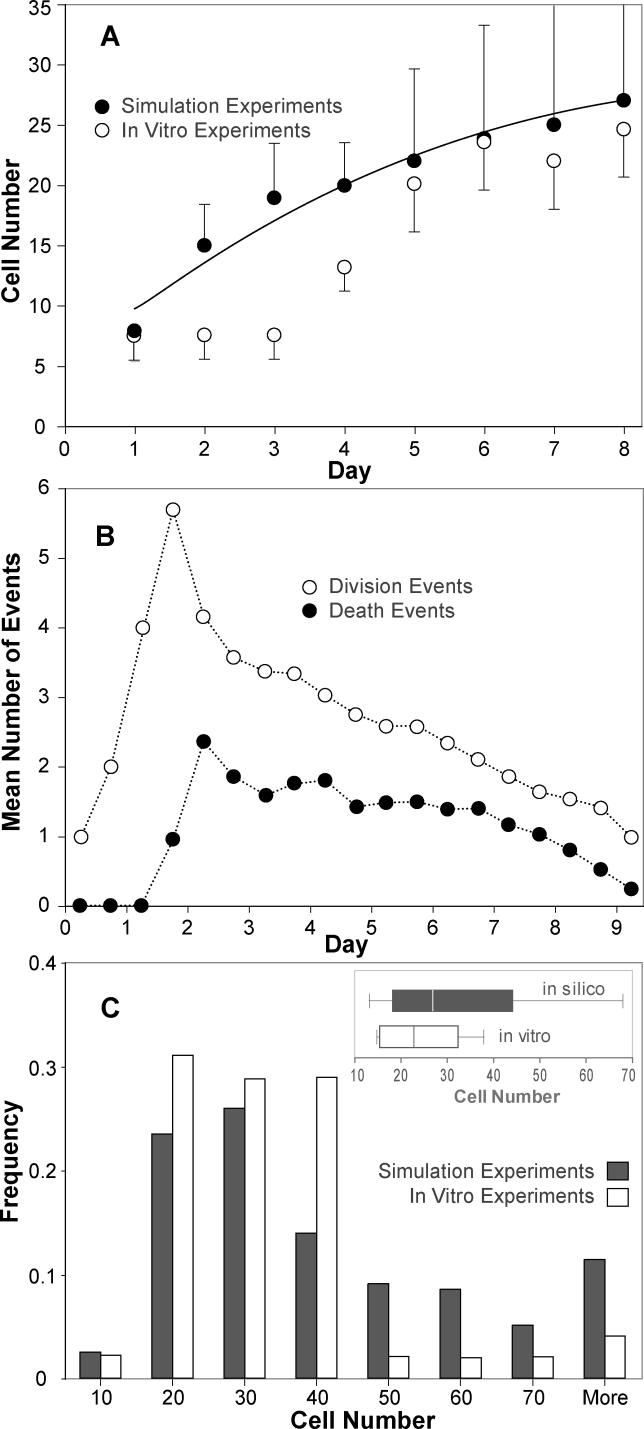
Analogue 2: Comparisons of In Silico and In Vitro Results Comparisons are presented of data from experiments in silico (*n* = 50), using Analogue 2, and in vitro data. A mean cell division time of 12.0 h is assumed. (A) Cell numbers per cyst in simulated embedded culture. In vitro cell number per cyst data was adapted from the cyst size time series in embedded culture provided in Figure 9 in [7], assuming an EC diameter of 10 μm and spherical hollow cysts. The bars indicate one standard error of the mean. (B) Average division event and death event counts at each simulation cycle. (C) The frequency distribution of cell numbers in vitro per cyst cross section after ten days in embedded culture are compared withthe frequency distribution of cell numbers per cyst cross section after 12 simulation cycles; black bars: simulation results; white bars: in vitro measurements. The former (in vitro) dataset was obtained by measuring MDCK cyst sizes at day 10 from Figure 2A in [24] (*n* = 44), assuming an EC diameter of 10 μm and spherical hollow cysts. The latter dataset was the result of *n* = 1,000 simulations. The insert shows the box plots for both datasets. The box centerline designates the median; the box designates the first through third quartiles; and the whiskers represent the 10% and 90% quantiles.

The next task was to confirm that the stable structures formed by Analogue 2 exhibit some of the PCs expected of cells exhibiting apical–basolateral polarity. Placement of a layer of cells on top of an existing monolayer of pcells (Figure 9, cycle 0“) resulted in a response that was much less dramatic (Figure 9, cycle 15) than that observed with Analogue 1. Inverted cysts of the type expected in suspension culture were present. This evidence helps make the case that Analogue 2 exhibits the expanded set of targeted attributes. What about in silico–in vitro similarity: how similar are the growth properties of Analogue 2 to those observed in vitro? In silico and in vitro experimental results are contrasted in Figure 10. The similarities are striking. Coupling this evidence with that above, we concluded that the expanded set of phenotypic attributes had been achieved.

### Models of EC Dysfunction

Important to the refinement of these analogues is increasing the in silico–to–in vitro overlap of abnormal as well as normal EC attributes. The importance of an axiom to normal behaviors can be examined by documenting the consequences of a significant change. Changing or electing not to use an axiom produces a new model. Contrasting the before and after behaviors helps us learn about the parent model. To better characterize the consequences of model changes, we studied the outcomes for the four simulated culture conditions in Figure 1.

**Figure 11:**
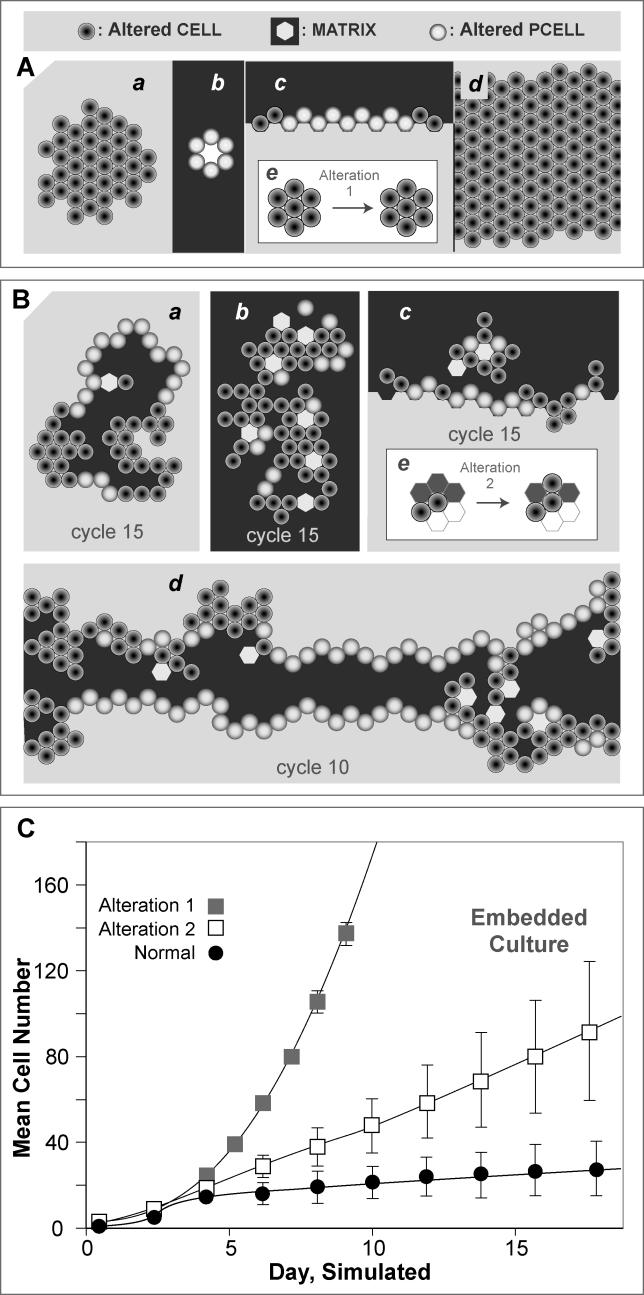
Consequence of Altering Two Axioms, 1 and 5 Growth characteristics that are a consequence of altering two axioms (1 and 5) controlling death and division are illustrated for four simulated culture conditions. As shown at the top in (A) and (B), cells have different shading than they do in Figures 8 and 9 because their behaviors are governed by a different set of axioms. Spaces are shaded as defined in Figure 8. The behavior of a pcell, when formed, was not influenced by the altered axioms. (A) Growth resulting from blocking cell death in Axioms 1 and 5 (Alteration 1). (B) Growth resulting from a simulated loss on an aspect of polarity caused by disrupting the directional cell placement in Axioms 7 and 8 so that daughter cells would be placed in any free space (Alteration 2). In both (A) and (B) the simulated growth conditions are as follows; **a**: simulated embedded culture; **b**: simulated suspension culture; **c**: simulated surface culture; and **d**: simulated overlay culture. In both (A) and (B) an example of altered axioms is provided by the insert **e**. (C) Cell and pcell growth is plotted for simulations in embedded culture that use either the normal axioms (Figures 3 and 7) or follow Alteration 1 or 2. The vertical bars are ± one standard deviation for 50 independent simulations.

Results from three distinct alterations to the axioms were studied. The first two are summarized in Figure 11. Alteration 1 was motivated by observations that inhibition of apoptosis results in a distinct morphology in 3-D embedded culture. Bcl-2, an inhibitor of apoptosis, has been overexpressed in MDCK cells [12], enabling the cells to survive in the absence of matrix. The result is evidence of the important contribution of cell death to cyst formation in embedded culture. Lumen formation in MDCK cell clusters that overexpress Bcl-2 is delayed. We simulated this intervention by not using Axioms 1 and 5: cells do not “die” in the absence of a matrix neighbor. The consequence, illustrated in Figure 11A, was behaviors that were distinct from those of unaltered Analogue 2 and consistent with the above in vitro experimental observations: in simulated embedded culture, cell clusters formed without lumens (Figure 11Aa), while continuing to form a normal, growth-arrested monolayer in simulated surface culture (Figure 11Ac). We also observed an increase in cell proliferation without growth arrest (Figure 11C) in simulated embedded culture. However, an important difference existed between these in silico observations and the above cited in vitro outcomes: arrest in cell cluster growth eventually occurred in vitro (with some lumen formation thereafter). There was no such arrest in silico, suggesting that in vitro processes other than apoptosis are likely contributing to lumen formation and arrest in cell cluster growth. Those processes have no counterpart in Analogue 2. The growth behavior of Alteration 1 also mimics the behavior of the cancerous mammary gland EC line MCF10A. In 3-D embedded culture, MCF10A cells form clusters without lumens. The similar behavior shown in Figure 11A reinforces the opinion that the MCF10A phenotype is due primarily to inhibition of cell death.

Alteration 2 was motivated by observations that cells in epithelial tumors appear less polarized [17]. Mutations in neoplastic tumor suppressor genes in *Drosophila* result in a loss of apical polarity and formation of large amorphous cell masses during larval development [18]. We simulated an aspect of loss of polarity by disrupting the directional cell placements specified in Axioms 7 and 8. We created an analogue in which we replaced Axioms 7 and 8 with altered Axioms (7′ and 8′); the latter directed that the daughter cell be placed in any free space (rather than adjacent to matrix). The characteristic patterns of growth that resulted are shown in Figure 11B. That change caused a unique in silico phenotype to develop, distinct from that observed for Alteration 1. A mixture of pcells and cells was observed in all four simulated culture conditions. That change was not the case for Alteration 1: it resulted in normal-appearing monolayers in simulated surface culture and normal-appearing cysts in simulated suspension culture: Alteration 2 did not. A comparison of growth rates for the two alterations documents the failure of growth arrest in both cases (Figure 11C). The mixture of simulated polarized and unpolarized cells (pcells and cells) resulting from Alteration 2 (Figure 11Ba–11Bd) resembles precancerous phenotypes found in the mammary gland, such as ductal carcinoma in situ, in which lumen formation occurs, and polarization remains, but there is some accumulation of ECs around the cyst wall, similar to Figure 11Ba [19,20].

**Figure 12:**
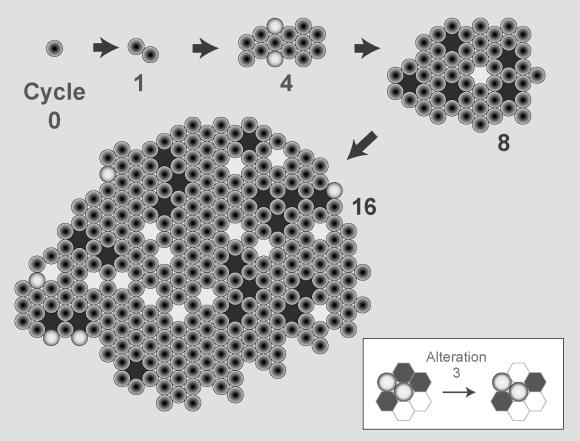
Consequences of Altered Matrix Production Altered matrix production causes loss of growth control in simulated culture conditions. Axioms 4 and 5 were replaced with a new one (an example of which is shown in the insert) that allowed for de novo matrix production in any environment consisting of free space and cell neighbors (Alteration 3). This sequence of illustrations of typical simulation outcomes shows the consequences of Alteration 3 on cyst formation in simulated embedded culture. The behavior of a pcell, when formed, was not influenced by the altered axiom, only the behavior of cells. As in Figure 11, cell shading is different to identify that an altered axiom set was used. Spaces are shaded as defined in Figure 8. At simulation cycle 0 a cell is added to a matrix-filled grid. At the conclusion of cycle 4, a small cluster had formed containing two pcells. By cycle 8 a distinct pattern of unstable growth was evident, with matrix- and free space–containing, lumen-like spaces within clusters of cells. That trend was still evident at cycle 16; the outer edge contained a few pcells. Thereafter, there was no growth.

Alteration 3 was motivated by a desire to study the potential consequences of aberrant matrix production on cell growth. However, we are not aware of a corresponding in vitro experimental system to which we can compare analogue behaviors. Because matrix can cause ECs to depolarize in vitro, we speculated that regulation of the direction of matrix production in silico might be an important feature to enable achievement and maintenance of the original four targeted attributes (the “normal” phenotype). To explore this idea, we liberalized matrix production by replacing Axioms 4 and 5 with an axiom that allowed for matrix production in any environment consisting of free space and cell neighbors. An example of that axiom is shown in Figure 12. We were interested in observing the organization of the components as the simulation progressed in the four simulated conditions, and determining whether growth stabilization would occur in any of the simulated environments.

In simulated embedded culture, Alteration 3 caused a third unique phenotype, distinct from those of Alterations 1 and 2 (Figure 12): cellularization and matrix production characterized the centers of growing clusters (Steps 8 and 16, Figure 12). Lumen formation and growth arrest failed to occur. We documented a hyperproliferative, cancer-like behavior solely due to less restricted matrix production. The results of these three sets of experiments illustrate the necessity and importance of designing analogues to exhibit capabilities 1–7 and 9.

## Discussion

The success of a model can be measured by how well it meets its intended uses. For this project, we sought models that would have two uses: simulation results mimic prespecified growth behaviors of ECs in four—not just one—in vitro culture conditions; and one of the models could serve as a foundational model. Both uses have been demonstrated.

A long-term goal is the construction of detailed simulation models—in silico analogues—of EC morphogenesis. The envisioned analogues are expected to be useful as a working (easily studied) hypothesis of how EC growth and morphogenesis occurs; as a resource of existing biological knowledge about ECs; and in optimizing designs of artificial tissues. To begin, we have focused our efforts on modeling the less complicated, constrained in vitro cell culture systems used in research. For that, a foundational analogue was needed along with a systematic process for continual study and refinement leading to analogues that are more detailed. We presented such an analogue—Analogue 1—and a descendent—Analogue 2. We characterized the extent to which Analogue 1 represents a prespecified set of EC behaviors. We present an iterative refinement process (illustrated in Figure 2) that was used to create Analogue 2. It is only slightly more complicated than its predecessor, and represents the same prespecified behaviors while avoiding an important Analogue 1 weakness.

### The Foundational Analogue

Several approaches were available for constructing an initial analogue. Cellular Potts models have demonstrated their ability to represent a range of cell morphological behaviors. However, a primary assumption was that morphogenesis is solely a result of differential adhesion. Experimental evidence has demonstrated that more processes are involved. For example, directed transport of solutes [21] and de novo production of basement membrane [22] are also important. We needed an approach that would allow for multiple aspects of EC behavior to be simulated as efficiently as possible.

The refinement process benefited from a modeling approach and framework that emphasized the nine capabilities listed in Materials and Methods. Analogue 1 proved to be sufficiently flexible: adaptations were easily made, and it was relatively simple to change assumptions and alter components to better match the targeted attributes, without requiring significant analogue reengineering. Using discrete interactions between components that are themselves discrete enabled analogue testing under a range of simulated growth conditions. This process is similar to the process of measuring the norms of reaction of genotypes in response to changes in the environment. Characterizing analogue behaviors under several growth conditions will be an essential feature of model refinement as it aids in identifying the consequences of analogue changes that may not be apparent under just one or two simulation conditions.

To construct Analogue 1 with as few assumptions as possible, it was essential to begin at the level of resolution for which the most comprehensive systemic information about EC behavior exists. A great deal of information has been acquired about the effect of environment on EC growth and behavior. This information confirms environment composition and configuration as important behavioral determinants. By iteratively combing results of experiments and then experimenting in silico, we acquired a set of axioms that realistically shaped cell behavior in four simulated environments. These axioms include consideration of phenomena such as adhesion preference and daughter placement preferences. In some cases, such as in the initial stages of embedded culture, ECs show a preference for adhesion to other ECs. In contrast, in surface culture and in later stages in embedded culture, they appear to adhere preferentially to matrix surfaces. Such preferences have been efficiently represented in the axioms through consideration of an individual's behavior within collections of cells in a particular environment (axioms 6, 7, and 8). By focusing on fundamental behaviors of ECs, we were also able to incorporate aspects of EC biology in addition to differential adhesion, such as matrix production (axiom 4), anoikis (axiom 5), and homeostasis (axiom 9).

Analogue 1 proved capable of mimicking in vitro experimental outcomes for four different simulated culture conditions, demonstrating that its small set of axioms is sufficient to represent a range of fundamental EC behaviors. The results support the hypothesis that the formation of multicellular epithelial structures is strongly influenced by environment composition and configuration. It also demonstrates that high-resolution analogues are not required to arrive at a reasonable approximation of characteristic systemic experimental outcomes. The results also support the notion that the decisional options available to each cell are tightly controlled and regulated. Finally, the results imply that normal growth and morphogenesis tolerate very little individual variability in cell behavior.

Axiom development was based in part on the results of simulation experiments as well as in vitro experimental observations reported in the literature. Because of the low resolution, there were only a few neighborhood arrangements for which there was no in vitro evidence on which to base an axiomatic outcome assignment. For example, refer to the top pcell example in Figure 7A. Imagine the center matrix replaced by free space. Faced with that neighborhood arrangement, how should the cell respond? Should it do nothing, replace that free space with matrix, divide and replace that free space with a daughter, take some other action, or select randomly from among options? There was no in vitro evidence on which to base a choice. In such situations, we took into consideration such factors as the differential affinity that ECs can exhibit towards matrix and towards other ECs (see Materials and Methods), and then compared simulation outcomes for the available options, and selected the one with outcomes closest to the targeted attributes. If the resolution had been higher, there would have been many more such uncertain situations: a resulting analogue mechanism would be less evidence-based and more modeler specified (speculative).

### Iterative Model Refinement

We used a four-step refinement process to arrive at Analogue 2. It proved capable of mimicking the same, targeted behaviors, as did Analogue 1, while successfully addressing limitations of the latter. Such models will have to be constructed in the face of sparse and non-uniform coverage of the system's possible behavior space. Because they are models, it is clear that they will never be complete. It is therefore important that they be continuously tested and refined in a process that challenges them with an expanding set of targeted attributes, either newly acquired or selected based on the existing literature evidence.

The adoption of apical–basolateral polarity by ECs is a fundamental event in the development of mature epithelia. The process plays a key role in morphogenesis, as knockouts of genes involved in this process in D. melanogaster cause epithelial tissues to proliferate abnormally. Axioms 3 and 6–9 (Figure 3) provide for polarized behaviors in silico: they are built-in. However, all Analogue 1 cells follow the same decisional process (Figure 4). A cell that used axioms 6–9 in simulation cycle *n – 1,* ignores that fact during simulation cycle *n*. Consequently, when specifically challenged, Analogue 1 exhibited a behavior inconsistent with known polarized behaviors (Figure 6). Following iterative refinement, Analogue 2 exhibited an improved, acceptable behavior (Figure 9): components were increased from three to four by including pcells. Outcome options were expanded by including pcell-specific behaviors. The improved performance of Analogue 2 reflects the known importance of apical–basolateral polarity.

Analogue 2 served as the starting point for in silico studies of dysregulated cell behavior (Alterations 1–3). The experimental results showed that two different changes in the tightly controlled axioms (Alterations 2 and 3) caused formation of abnormal yet similar looking structures to form. Likewise, aberrant matrix production Alteration 3 led to dysregulated growth. If Analogue 2 axioms do have an EC counterpart, then these observations can motivate hypotheses that can be tested in vitro when the potential knowledge gained merits doing so. For example, under some EC growth conditions, aberrant matrix production can cause dysregulated growth; and while abnormal EC masses formed in vivo and in vitro may appear similar, the underlying causes may be distinct.

By extending the targeted attributes list by one, we increased the complexity of the analogue. That occurrence reinforces the importance of the nine capabilities listed in Materials and Methods, and stresses that, when following the iterative refinement method in Figure 2, one needs to take modest-sized, carefully selected steps.

### Limitations of Analogue 2

Selecting and targeting a small set of phenotypic attributes can be viewed as a limitation. There are many attributes, not included in the targeted set, which Analogue 2 fails to mimic. When any of these excluded attributes is added to the targeted set, as described in Materials and Methods and in Figure 2, Analogue 2 becomes invalid. It is valid for the original target set of attributes, but invalid for the expanded set. When adding a new attribute to the target set, we preferred behaviors that are mechanistically close to those of the current analogue and where there is an expectation that they can be realized through modest extension of the model. We present three examples of attributes that we have considered for inclusion in an expanded targeted set. First, it has been observed that when inverted MDCK cysts, formed in suspension culture, are transferred to an embedded culture, inversion of cell polarity takes place with minimal cell proliferation or migration. The matrix within the cyst is degraded, and cell surface marker proteins relocate between the lateral and basolateral surfaces of the ECs, inverting their apical–basolateral polarity [23]. We simulated that experiment using Analogue 2 to observe the consequences. An inverted cyst was placed in simulated embedded conditions and the simulation was started. After several simulation cycles involving growth and change, stable structures formed: cysts with a free space interior typical of cysts grown from single cells in simulated embedded culture. However, unlike in vitro, cyst formation required considerable cell proliferation, and the roles played by matrix appeared not to be analogous to that in vitro.

A second example is that de novo matrix production is restricted to Axiom 4: when a cell has only one cell neighbor and no matrix neighbors. It enables normal cyst formation in simulated suspension culture. However, in wet-lab suspension cultures, aggregates of more than two MDCK cells occur and form normal cysts [7]. Attempts to simulate this behavior using an altered Axiom 4 were successful for simulated suspension cultures, but abnormal growth resulted in simulated embedded cultures: cells found on the inside of expanding cysts but not in contact with matrix would produce matrix de novo, resulting in uncharacteristic structures. Those results suggest a hypothesis: either ECs residing on the inside of expanding cysts in vitro have a means to regulate matrix production that is specific to that condition, or that matrix-degrading components are secreted into the luminal spaces by ECs that already have matrix contact, or both. A more refined analogue may provide a solution. The third example is that lumen formation in embedded cultures does not always involve apoptosis. For some MDCK cells, there appears to be processes other than apoptosis that contribute to lumen formation [7].

Here are three examples of the more complicated behaviors that will be candidates for targeting by descendents of Analogue 2. 1) Growth of ECs in embedded cultures having increasing densities of matrix is associated with a transition from lumen formation and arrested cyst growth to proliferation and growth of lumen-less cell masses. 2) The presence of other cell types such as myoepithelial cells can influence EC growth (e.g., mammary ECs). 3) Introduction of a growth factor such as HGF induces a multistep branching response in vitro.

Selecting and prioritizing such attributes for inclusion in the targeted set is an important part of the systematic analogue refinement process shown in Figure 2. More detailed phenotype reporting from experimental studies will be helpful in accelerating analogue refinement. In vitro experiments may also be needed for the sole purpose of analogue invalidation (or validation).

### Moving Forward

The axioms and their application comprise the in silico mechanism. We have not offered a cell biology explanation for the axioms, because we expect that to unfold as a natural consequence of iterative analogue refinement. The axioms used are high-level, low-resolution placeholders for more detailed representations of the actual complex mechanisms driving behaviors. Use of axioms precludes direct connections to the abundant, detailed intracellular information that is available. We need a mapping between the elements of Analogues 1 and 2 and elements of the biological system. Within limits, each EC is an independent, autonomous actor in its in vitro environment. Each cell has innate capabilities and functions that have been documented separately and collectively by experimentation. These capabilities contribute to the overall mechanism. An operating hypothesis has been that internal coordination and control is tight: sufficiently so that an observer may infer that behaviors are following innate mandates, presumably directed by the cell's genome, its history, and the spatiotemporal organization and interaction of its components. For each axiom in Table 1, the specific functions checked are hypothesized to be essential for application of that axiom. Together, they provide a tentative mapping between elements of Analogues 1 and 2, and those of the referent ECs.

**Table 1:**
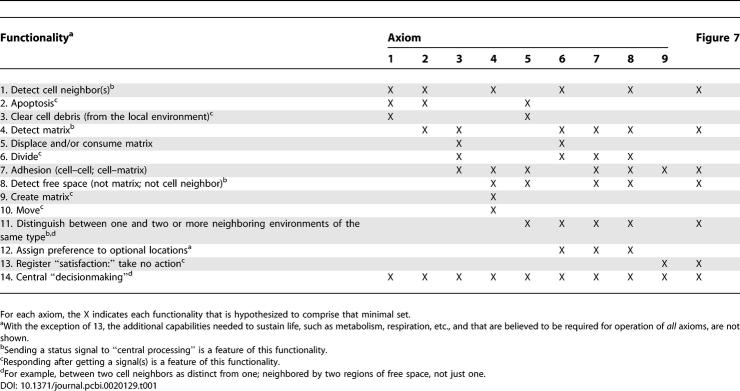
A Mapping of the Axioms Listed in Figure 3 to Several MDCK Cell Capabilities

Why not design and build a mechanism that utilizes the interaction of each of the capabilities listed in Table 1 rather than starting with the more abstract set of axioms? The latter approach provides the simplest method for building a foundational analogue and for testing the hypothesis that the targeted attributes can be achieved by a mechanism that senses, responds to, and can create three primary environment components. By first focusing on axioms that specify an outcome given a precondition, we were not weighted down and distracted by our knowledge of the details of each of the capabilities.

Refer to Analogue 2 as an AM. If our operating hypothesis is reasonably correct, then we should be able to also create at least one additional analogue in which each function in Table 1 is specifically represented. Refer to that as an FBM. The more detailed, independent processes giving rise to each function within a FBM would need to be coordinated in ways that, for specific inputs, the behaviors of the AM and FBM become essentially indistinguishable over a variety of experimental conditions, thus establishing cross-model validity. Extending the coverage of the AM (Figure 2C) and developing an FBM are two different directions that this research could take. An advantage of the former is that it may be easier and faster to sequentially extend Analogue 2 to account for an expanded set of targeted attributes. An advantage of the latter direction is that by designing FBMs to be modular, functionality can be represented by a separate, interactive component (that maps to recognizable cell components) within each cell. Both allow for instantiation and testing of the conceptual model described in Materials and Methods.

An advantage to parallel pursuit of both model types is that the composite FBMs can be validated, or not, against both the set of targeted attributes, and—*importantly—*the validated behaviors of its AM counterpart. In theory, such a validation process can be automated. Another advantage is that incrementally, more of the added mechanistic detail will map logically to more of the molecular detail that is currently so abundant in the literature. It becomes easy to see that this dual path approach can lead to collections of analogues that link molecular level events through multiple higher levels of instantiated processes to system-level phenotypic attributes, and that achievement is one of the core goals of computational systems biology.

## Materials and Methods

Our “middle-out” approach to biological system modeling is motivated by aspect-oriented software development. We adapted the latter in arriving at our working definition of middle-out modeling: specify the biological features under study (e.g., achieving a particular arrangement of cells and environmental components, given a particular initial condition). Each feature becomes an aspect of the model (the software device). The resulting models become functioning analogues, in our case, software analogues, of in vitro biological systems.

For this project, our goal is to implement a plausible in silico mechanism that can realize a targeted set of in silico attributes that are acceptable matches to the target set of already studied, already quantified, in vitro phenotypic attributes.

We first pick a set of properties, p_1_, p_2_, . . ., p_j_ (within a hyperspace of mechanistic properties)—around the level of interest (e.g., around in vitro cell growth and structure formation in the case of MDCK cells). An example of a property on which we focused was that matrix added atop a stable epithelial monolayer induces a proliferative response and lumen formation. We then ask what software device can be implemented to realize p_1_? How can we realize p_2_, etc.? Next, we ask, how can p_1_–p_j_ be realized all at the same time, using the same software device? The in silico properties and the device we create to realize p_1_–p_j_ form a kind of simulation kernel—a foundational analogue—from which we expect to expand outward (up, down, and even sideways) to establish a reductive hierarchy and identify important principles.

The goal of our middle-out approach is establishment of plausible reductive hierarchies between lower level mechanisms and higher-level phenomena by growing useful, more detailed in silico analogues from an arbitrary foundational analogue.

### Creating an analogue phenotype.

A long-term goal is to develop validated simulations of mammalian ECs growing in a range of cell culture conditions. They will have many behaviors, properties, and characteristics that mimic those of referent cell culture systems. Stated differently, there will be similarities between the phenotype of the cell line being studied and the “phenotype” of the simulated cells in the model (Figure 2). The expectation is that increasing similarity in phenotype will require, and can be achieved through, similarities in generative mechanisms. At that stage, the model will have become a scientifically useful analogue of the referent cell line. In this report, an analogue is a model of an EC line capable of mimicking a set of targeted growth characteristics. An in silico growth system (in silico system for short) is a model of an in vitro cell culture system (cells, media, container, atmosphere, etc.) that starts with one or more cell models. There will be a need for a hierarchy of models. An in silico component of an in silico growth system is itself a model of a corresponding feature or component of the referent system.

The envisioned relationship between an analogue and its in vitro cell culture referents is illustrated in Figure 2. Pictured are two overlapping (but not intersecting) sets. One set contains the results of experiments that measured specific phenotypic attributes, properties, or characteristics of in vitro cell culture systems (Figure 2A, large circle). The other, smaller set contains the results of simulation experiments that measured corresponding phenotypic attributes, measurable properties, or characteristics of the in silico system. When the experimental measures between the two sets are similar, such as cyst formation in a particular environment or growth rate, then there may also be useful similarities in the generative mechanisms of the two systems, and these can be explored by iterative testing and refinement of the analogue coupled with related wet-lab experiments. Because we cannot expect these sets of measures to overlap completely, the analogues will be able to generate behaviors for which there are no in vitro counterparts: they are biologically impracticable. There will also be conditions under which there is an in vitro counterpart, but the wet-lab experiments to measure that behavior have not been done. The framework and approach used here allow for rapid exploration of possible generators of behaviors under many different conditions. Observations on behavior similarity can be used simultaneously to eliminate invalid model features and identify gaps in our knowledge of the in vitro experimental system.

The strategy that we have learned and have used to develop Analogue 1 from its more impoverished predecessors has proven equally effective in moving forward to Analogue 2, and is expected to be effective in developing even more improved, informative, realistic, and heuristic in silico devices. The first step in transitioning from a current analogue (e.g., Analogue 1) to an improved analogue (e.g., Analogue 2) was to more thoroughly document the in silico PCs of the partially validated, earlier analogue. By doing so, we improved insight into its strengths and weaknesses. The next step was to obtain increasing overlap of measures of in vitro attributes. That was done systematically by iteratively revising together the hypothesis (that the current analogue can acceptably simulate the set of targeted attributes) and the analogue, as follows.

1) Select an additional in vitro property or characteristic that is related to those already in the target set, and for which wet-lab experimental observations are available. 2) Add that attribute to the current targeted set. 3) Determine if addition of the new attributes causes the already accepted analogue (e.g., Analogue 1) to invalidate, and if so, why. If not, repeat step 2. 4) Revise the model iteratively, possibly by adding mechanistic detail, until the measured phenotypic attributes of the revised analogue (e.g., Analogue 2) are sufficiently similar to the expanded set of targeted attributes. This four-step approach is illustrated in Figure 2C.

Our approach to achieving such an analogue was influenced by the following nine additional capabilities (and specifications). We reasoned it would be essential for the envisioned analogues to exhibit these capabilities to achieve our long-term goal.

1) *Morphogenesis.* Analogues (including cells, pcells, and future types) are capable of accurately representing the targeted, characteristic patterns of growth and morphogenesis typical of ECs grown in different in vitro environments. 2) *Mapping.* There is clear *mapping* between in silico and referent system components because analogue behaviors and observables are designed to be consistent with those of ECs grown in vitro. 3) *Turing test.* Measures, properties, and characteristics of in silico growth are, to a domain expert (in a type of Turing test), experimentally indistinguishable from corresponding in vitro measures; this requires that in silico systems must be suitable for experimentation. 4) *Transparent.* An in silico system and its components must be *transparent*. The details of a simulation, as it progresses, needs to be visualizable, measurable, and comparable to those of the referent. 5) *Articulate.* The components *articulate*. Components, analogous to their referent counterparts, are designed to interact. It must be easy to join, disconnect, and replace components. 6) *Flexible.* It must be relatively simple to change usage and assumptions, or increase or decrease analogue or in silico system detail in order to meet the particular needs of an experiment, without requiring significant reengineering of the model. 7) *Reusable.* System components (including analogue “cells”) must be *reusable* for simulating EC behaviors in different experimental conditions, in vitro and in vivo, in the presence and absence of treatments and interventions. 8) *Adaptable.* Analogues must be constructed so that they can function as components of tissue models. 9) *Discrete interactions.* To enable the above capabilities, the analogues, in silico system components, and their framework must use *discrete interactions*.

The first step was to create a reasonably simple foundational analogue that serves as precursor to the envisioned, more capable analogues, one that can generate behaviors that mimic a target set of in vitro phenotypic attributes. Analogue 1 is the foundational analogue.

### Design of Analogue I.

*Components.* Observations of EC growth and morphogenesis in diverse in vitro culture conditions were used to inform the design and construction of Analogue 1. Motivated by observations that ECs respond to the presence of matrix, neighboring cells, and the loss of adjacent free surface, we began by limiting components to three types: cells, matrix, and free space. We assumed that cell actions would be influenced by the relative locations and types of adjacent components. Although it is well known that ECs growing in culture can differentiate and polarize, there is no evidence that the basic mechanism (and logic) that is responsible for fundamental EC characteristics is also changing. Consequently, we assumed that, in terms of the mechanisms of interest, all ECs are essentially identical and that current actions, being primarily controlled by apparent mandates, are independent of past actions. Although there is ample evidence that the matrix produced by ECs has somewhat different physical properties and composition than the matrix typically used for in vitro cultures, we assumed that for the course behaviors required of Analogue 1 (e.g., die, divide, etc.) there is no current need to distinguish between the different types of matrix. Everything that is neither matrix nor cell, such as suspension culture media and luminal contents, is conflated and represented by one component: free space.

*Environment.* We needed a model environment in which to place the above components, and a strategy for defining relationships among them. For Analogue 1 we elected to keep it simple and use a 2-D hexagonal grid, with one component assigned to each grid position. With this representation, each cell is neighbored by six grid positions. Other strategies for specifying the environment's structure and component interactions are available, including 2-D square grids, 3-D grids, and randomized networks. We opted to use a hexagonal grid because it provides 64 distinct and nonsymmetric configurations for three different components relative to the center cell. We speculated that this environment representation would provide ample variety to achieve overlap with a number of attributes from in vitro cell culture systems. All grid locations are the same size. We make no specific assumptions about dimensional mappings between a unit of cell, matrix, or free space and specific measurements in a cell culture.

*Cell behavior.* Only cells can act. We limited the number of actions available to a cell to four. A cell can “die,” “divide” (duplicate itself), generate matrix, or do nothing. When a cell “dies,” it is removed from the simulation and replaced with free space. When it divides, a new cell is placed at an adjacent position. A cell generates matrix by moving to and replacing a neighboring free space, and simultaneously placing matrix in its vacated position. When an EC is not engaged in any comparable action, it is engaged in other functions that may not affect morphogenesis but are important to other aspects of its function and survival such as relative migration (small changes in position relative to adjacent components), and the de novo creation of cell and luminal spaces. All of these activities are represented in the analogue by “do nothing.” Relative migration was treated as being below current resolution and so is not represented. The de novo creation of luminal spaces by ECs in vitro is an important consideration for future descendents of Analogue 1, and is discussed later.

We assumed that each action taken by a cell in vitro requires that one or more preconditions have been met. We imposed that requirement on Analogue 1: each action requires that a precondition be met. For simplicity, we limited the set of preconditions available to a cell to those that can be specified by the nature and relative arrangement of the six adjacent components. The task then became this: for each of the 64 unique component configurations, we should rank-order the four action options from most likely to least likely, and then implement the result as a simulation using the highest ranked of the four action options. The procedure that follows is an abridged version of the development effort. It was readily apparent that for a subset we could use experimental observations of EC behavior reported in the literature to confidently guide the ranking. Despite the low resolution and the availability of considerable experimental evidence, for a few cases there was no experimental evidence supporting any particular ranking. For those cases, a ranking was assigned after taking into consideration several factors. They included the differential affinity that ECs can exhibit towards matrix and towards other ECs, the requirement of matrix neighbors for resistance to anoikis, and preference for cell division to occur in directions that minimize resistances imposed by matrix. We then had an action option ranking for each neighborhood arrangement. To enable a preliminary simulation, we arbitrarily assumed that, given the precondition of a specific neighborhood arrangement, the top ranked action would always occur. We then clustered the 64 neighborhood arrangements by assigned action. Within each cluster, we looked for common features within the component arrangements that would allow one axiom to be applied: if a specific precondition is met, then a specific action will follow.

Initially, there were many axioms. They enabled exploratory simulations to refine cluster identification and membership, and identification (from the early axioms) of possible chains of inference a cell might follow to be a member of a targeted stable structure. That iterative process leads to the terminal set of axioms in Figure 3 and the chain of inferences sketched in Figure 4. Analogue 1 implements that simple logic. It is an inference engine and is deductive. The implementation is observed as the interaction of components, thereby enabling specific behaviors to emerge. Figure 4 shows part of the core mechanism (logic) of the analogue device: the set of inferences that might apply (for a given cell) and the inferential flow of the analogue from premises (the type and arrangement of components at the end of cycle *n – 1*) to conclusion (the type and arrangement of components at the end of cycle *n*).

*Execution.* It takes a significant amount of time for cells to divide, to generate matrix, and to die. The time for mature adhesions to develop between cells, and between a cell and matrix, is on the order of several hours. The time for cell death and division to occur is longer, on the order of 12–24 h or more, depending on culture conditions. We set one simulation cycle to represent a time duration sufficient to cover average cell death and division events.

Model execution is controlled as follows: at the start of the simulation, each cell is scheduled on a master schedule. At the first simulation cycle, the schedule is executed in random order. Each execution is a step within the simulation cycle. When a cell divides, the daughter cell is placed immediately in the environment. The daughter and parent are then scheduled for the next simulation cycle. If a cell does nothing, it schedules itself for the next simulation cycle. If a cell dies, it is immediately removed from the schedule and from the model environment. The preceding procedure assumes that in vitro the cell's operational interface accepts stimuli at nearly random intervals, processes them, and immediately responds with the appropriate behavior. Because a simulation cycle is the lowest level of time resolution, no correspondence is assumed between the chain of inferences sketched in Figure 4 and actual events that may be occurring in vitro. When the time resolution is increased, additional in vitro events will need to be represented.

We used MASON (http://cs.gmu.edu/~eclab/projects/mason) to implement the models. MASON is a small, optimized, and extensible Java-based simulation toolkit based on the Swarm simulation package; it enables simulation of discrete event models and includes a random number generator based on the Mersenne Twister algorithm. For data export, we used the JFreeChart library.

### Experimental assessment of Analogue 1.

Four simulated environments were constructed to represent the referent environments. Each consisted of a 2-D hexagonal grid, typically 100 × 100. Just one of three different types of components was assigned to each grid point. The properties of the environment, and thus the in silico system, were determined by the components assigned to each grid space prior to simulation. 1) An in vitro surface culture was represented by a grid in which there is an upper region of free space, and a lower region of matrix. To initiate the simulation, a single cell replaced a free space in the middle, at the cell–free space interface. 2) An in vitro embedded culture was represented by a grid in which matrix was assigned initially to all locations. To initiate the simulation, one or more cells replaced matrix at the center of the grid, for example. 3) An in vitro suspension culture was represented by a grid in which free space was assigned initially to all locations. A simulation was initiated by having two or more cells replace free space. 4) Finally, simulation of an in vitro overlay culture (also called sandwich culture) started with a single horizontal monolayer of cells bordered above and below by a region of matrix.

The seeds of random number generators were changed randomly for each simulation. Consequently, each simulation produced unique results. To capture a representative set of cyst shapes for analysis, experiments in simulated embedded culture, typically 100–200 cycles each, were repeated up to 1,000 times. Images of each completed simulation were collected for subsequent comparison from all four simulated culture conditions. For analyses of cyst growth in simulated embedded culture, simulations were run 50 times or were stopped shortly after cyst growth arrest. Screen captures of each simulation were saved. Cell component numbers at each simulation step were also saved.

### Challenging Analogue 1.

Although Analogue 1 allows for polarized responses of cells to the environment, the model assumes that cell behavior does not change. In vitro, there is evidence that EC behavior in a stabilized surface culture is different from that during growth and formation of that monolayer. In the former case, the cells exhibit apical–basolateral polarity that has resulted in a redistribution of cell surface molecules and production of a glycocalyx at the apical surface. This specialization of surface characteristics is believed to help account for fully differentiated cells being relatively unresponsive to apical surface contact by other cells. With this information in mind, as described earlier in the Creating an Analogue Phenotype section, we added a new attribute to the list of targeted attributes: once a structure or monolayer of simulated cells has stabilized, it should exhibit behaviors expected of fully differentiated, polarized cells. For example, it should be less responsive to apical contact with another cell than it would have been during growth (when it was not fully differentiated). We conducted a simple simulation experiment to document the behavior of a stable Analogue 1 monolayer (which is expected to mimic a condition in which apical–basolateral polarization has occurred) to an intervention of freshly added cells. After a stable monolayer had formed, the simulation was stopped. A layer of cells was added on top. The simulation was started. Images of the system were saved at each simulation step. Uncharacteristic proliferation was observed (Figure 6). That uncharacteristic proliferation invalidated Analogue 1 (see Figure 2).

### Design of Analogue 2.

To create a new analogue, Analogue 2, that would exhibit the expanded set of targeted attributes, including an appropriate response in the experiment described above, several options were considered. The following describes the development that led to Analogue 2. The variety of differentiation characteristics that become evident during growth and development of stable structures was arbitrarily divided into two classes. The class that is most abundant during early growth is represented by the cells of Analogue 1. The more differentiated, polarized cells that characterize stable cysts and monolayers are represented by a new component called pcells. Of the 64 neighborhood arrangements for Analogue 1, the three on the left side of Figure 7A were identified as characteristic of cells in stable structures in vitro. A new axiom was implemented (Figure 4, lower left): when any one of these preconditions is met, a cell-to-pcell transition occurs within that simulation cycle. Under appropriate conditions in vitro, such as in an overlay culture, polarized ECs de-differentiate. Under other conditions, they do not. We needed to specify pcell neighborhood arrangements that would cause simulated de-differentiation and those that would not (Figure 4, lower right). To do so we relied on the available experimental evidence and followed a protocol very close to that described under Design of Analogue I. Given the three arrangements in Figure 7A, and keeping the pcell and its adjacent “cell” neighbors (they could be either cell or pcell) in place, we identified 28 possible neighborhood changes that could occur between one simulation cycle and the next (Figure S6). We segregated them into two groups based on the in vitro experimental evidence. We identified 11 that would not likely stimulate de-differentiation. Several of the other 17 were known to stimulate de-differentiation. For Analogue 2, we arbitrarily decided that all 17 would do so. The 17 are easily subdivided into three groups to which the additional new axioms in Figure 8A apply.

### Experimental assessment of Analogue 2.

*Comparison with Analogue 1.* The method of Figure 2 requires that a candidate, improved model must exhibit the prior set of targeted phenotypic attributes (in this case, the set of targeted attributes to which Analogue 1 validated, as in Figure 5) while adequately overcoming the invalid behavior that motivated the revision. The behavior of Analogue 2 was studied under each of the same four simulated culture conditions used for Analogue 1. Experiments were conducted to compare in detail the behaviors of Analogues 1 and 2. Each analogue was simulated 1,000 times for 100 simulation cycles. The mean and standard deviations for “cell” numbers, type, division events, and death events were calculated for each cycle.

*Validation against the new phenotypic attribute.* The experiment described above under Challenging Analogue 1 was repeated using Analogue 2. The initial, stable monolayer was 100% pcells; cells were then added as a layer above the pcells and the simulation was restarted. The simulation had stabilized prior to simulation cycle 15.

*Altering axioms.* An alteration to Analogue 2 caused by changing or failure to use an axiom will change behavior whenever the precondition to which that axiom applies is present. We explored the consequences of three such alterations in each of the four simulated culture conditions. Knowing death is essential to development of characteristic EC structures, we studied the consequences of failure to use Axioms 1 and 5, called Alteration 1. A key feature of Axioms 7 and 8 is conditioned placement of the daughter. Alteration 2 eliminates that condition in both axioms. Aberrant matrix production may be a factor in EC dysfunction. Alteration 3 replaces Axioms 4 and 5 with one that mandates matrix production whenever the local neighborhood consists only of free space and cell(s).

The software used, along with executable applets, is available at https://128.218.188.102/growthmodel/index.html.

## Supporting Information

Figure S1 Preconditions for Application of Axioms 4–7 and 9 Preconditions (local neighborhood composition and arrangement) are identified for application of Axioms 4–7 and 9.
Axioms 1–3 mandate cell behavior in matrix-, free space-, and cell-only environments; a study of neighborhood permutations was not needed. Permutations of two-component neighborhoods, relative to the center cell, were examined. White hexagon: matrix; black: free space; gray: cell.
Axiom 5 mandates behavior for all neighborhoods comprising cells and free space: the center cell dies when any one of these preconditions is met.
Axiom 6 mandates behavior for all neighborhoods comprising cells and matrix: a daughter is created; it replaces any neighboring matrix except those marked x.
Axiom 7 mandates behavior for all neighborhoods comprising free space and matrix: a daughter is created; it replaces any neighboring free space except those marked x.
Axiom 9: there are just two arrangements of two-components neighborhoods for which Axiom 9 applies.
(185 KB TIF)

Figure S2 Preconditions for Application of Axiom 8 Preconditions (local neighborhood composition and arrangement) are identified for application of Axiom 8. Our assumption is that in vitro, a cell must be polarized in order to apply Axiom 8.
(A) Step (simulation cycle) *n – 1*: the 35 possible neighborhoods (of center cell) comprised at least one cell, one matrix, and two free spaces. White hexagon: matrix; black: free space; solid gray: cell. There are 87 free space options for daughter placement.
(B) Step *n*: gradient shaded hexagon: acceptable positions for daughter cell placement; black hexagon with white x: unacceptable position for daughter cell placement; 55 of the 87 daughter placement options are allowed; note that for each of the 55 allowed options, the parent and daughter cells maintain matrix contact (simulating maintaining matrix attachment) during and after division. A daughter cell placed in any one of the 22 excluded locations would lose the matrix contact had by the parent.
(358 KB TIF)

Figure S3 Additional Attributes (Discovered during Experimentation) That Were Not among Targeted Attributes (Growth in Presence of matrix Gaps, Surface Repair, and Cyst Joining) (A–C) Simulation of monolayer formation by Analogue 1 in the presence of matrix gaps (top); the outcome (bottom) depends on the nature of the matrix gap.
(D) Demonstration of surface repair using Analogue 2 (results were the same using Analogue 1). Left: cells flagged as “dead” in a stable cyst have been removed. Middle: repair progress after several additional simulation cycles. Right: repaired, stable cyst.
(E) A simulation of cyst joining using Analogue 2 (results were similar using Analogue 1). Left: simulated cystogenesis adjacent to a stable cyst. Middle: joined cyst prior to stabilization. Right: stabilized cyst (all pcells) after joining.
(200 KB TIF)

Figure S4 Further Analysis of Analogue 2 Outcomes in Simulated Embedded Culture (A) Examples of stable cyst structures formed in a simulated embedded culture.
(B) A contour plot; 200 stable cysts were formed in simulated embedded culture. The center grid location (x) for each was identified; the contour plot shows the probability of encountering a cell at 1, 2, 3, . . . grid spaces from that center.
(314 KB TIF)

Figure S5 Comparisons of Growth Properties of Analogues 1 and 2 in Simulated Embedded Culture (A) Mean cell numbers per simulated day.
(B) Mean cell division and death events.
(C) Frequency distribution of cell numbers per stable cyst.
In each case, the results from Analogue 2 included both cells and pcells; one simulation cycle = 12.0 h, *n* = 1,000.
(93 KB TIF)

Figure S6 Selection of Environment Permutations That Lead to Transition from a Pcell to a Cell
The shading is as described in Figure S1.
Top: relative to the center cell, each of these 17 neighborhood arrangements is a precondition for transition from a pcell to a cell.
Bottom: each of these 11 neighborhood arrangements is a precondition for remaining a pcell into the next simulation cycle.
(164 KB TIF)

## Abbreviations

AM: axiomatic model
EC: epithelial cell
FBM: functionality-based model
MDCK: Madin-Darby canine kidney
PCs: properties and characteristics
